# Intertidal insects associated with halophytic *Suaeda* (Amaranthaceae) in Japan: a case study in Saga, northern Kyushu

**DOI:** 10.3897/BDJ.10.e79184

**Published:** 2022-04-28

**Authors:** Akihito Kita, Ayman Khamis Elsayed, Makoto Tokuda

**Affiliations:** 1 Faculty of Agriculture, Saga University, Saga, Japan Faculty of Agriculture, Saga University Saga Japan; 2 Saga Prefectural Space and Science Museum, Takeo, Japan Saga Prefectural Space and Science Museum Takeo Japan; 3 The United Graduate School of Agricultural Sciences, Kagoshima University, Kagoshima, Japan The United Graduate School of Agricultural Sciences, Kagoshima University Kagoshima Japan

**Keywords:** halophyte, insect fauna, intertidal zone, *Suaeda*

## Abstract

In contrast to a great diversity in insects in terrestrial and freshwater ecosystems, few known species have adapted to inhabit marine environments. In this study, we surveyed insects associated with halophytic plants of *Suaeda* (Amaranthaceae) distributed in intertidal zones, in northern Kyushu, Japan. On four Japanese native species of *Suaeda*, we found insects belonging to five orders and 18 species. Amongst them, the genus *Clanoneurum* (Diptera: Ephydridae) and *Coleophoradeviella* (Lepidoptera: Coleophoridae) were newly reported from Japan; and Orthotylus (Melanotrichus) parvulus (Hemiptera: Miridae) was newly recorded from Kyushu. The seasonal occurrence of several insects on *Suaeda* is reported.

## Introduction

Insects have achieved a great diversity in terrestrial and freshwater ecosystems, but they seldom inhabit marine environments (e.g. Cheng 1976, Ward 1992). Only some insect taxa are known to adapt to intertidal zones and very few in neritic and oceanic environments (Ward 1992, Ikawa et al. 2012, Kaiser et al. 2016).

In intertidal zones, Coleoptera are the most dominant group in terms of the number of genera reported and followed by Diptera, Collembola and Hemiptera (Ward 1992). These insects exhibit various adaptations to seawater, such as plastron respiration under seawater and behavioural avoidance of submergence (Hinton 1976, Ward 1992). Adaptive mechanisms of intertidal insects to marine habitats are interesting study subjects to understand their survival strategies in harsh environments for many insects.

Some herbivorous insects are associated with halophytes growing in the intertidal zones (Foster and Treherne 1975, Dorchin 2001, Dorchin and Freidberg 2008, Cho et al. 2015, Elsayed et al. 2015). As herbivory critically affects some plant community structures in intertidal zones (Rand 2002, Pennings et al. 2007), further intensive studies of herbivores are important to understand their role in determining plant community structures in intertidal zones.

The genus *Suaeda* (Amaranthaceae) consists of approximately 100 species and most of them grow in coastal areas and tidal wetlands (Yonekura 2017). Several studies have reported insects on *Suaeda*, such as a psyllid (Hemiptera: Sternorrhyncha) associated with *Suaedajaponica* Makino in Korea (Cho et al. 2015); gall midges (Diptera: Cecidomyiidae) in the Holarctic Region and South America (Kieffer 1909, Kieffer and Jörgensen 1910, Felt 1918, Mamaev and Mirumian 1990, Dorchin 2001, Dorchin and Freidberg 2008, Skuhravá et al. 2014, Elsayed et al. 2015) and their hymenopteran parasitoids (Gates et al. 2018), a leaf beetle in North America (von Groll et al. 2022) and lepidopterans in various localities worldwide (Paik et al. 2013, Adamski et al. 2018, Karisch et al. 2020, Budashkin and Bidzilya 2021). However, information on insect fauna associated with *Suaeda* is still fragmental and comprehensive studies have never been conducted as far as we know.

The area facing the Ariake Sea in Saga Prefecture has the largest mudflats in Japan, owing to the largest tidal range in the country, which provides a suitable habitat for halophytes including *Suaeda* (Henmi et al. 2017, Tokuda 2019). In the present study, we periodically surveyed the insect fauna associated with four *Suaeda* species in Saga, Japan and examined the seasonal occurrence of several dominant insect species to reveal their ecological aspects.

## Materials and Methods

### Study plants

Four native species of *Suaeda* are known to be distributed in Japan (Yonekura 2017): *Suaedaglauca* (Bunge) Bunge distributed in Honshu and Kyushu, Japan as well as in eastern Siberia, Ussuri, Mongol, China and Korean Peninsula; Suaedamaritima(L.)Dumort.subsp.asiatica H. Hara distributed widely in East Asia including Honshu and Kyushu, Japan; *Suaedamalacosperma* H. Hara distributed in western Honshu and Kyushu, Japan, as well as in Korean Peninsula; and *S.japonica* distributed in northern Kyushu, Japan and Korean Peninsula. They are all annual plants (Yonekura 2017). We investigated insects associated with all four native *Suaeda* species in this study.

### Periodical investigations

**Figure 1.**
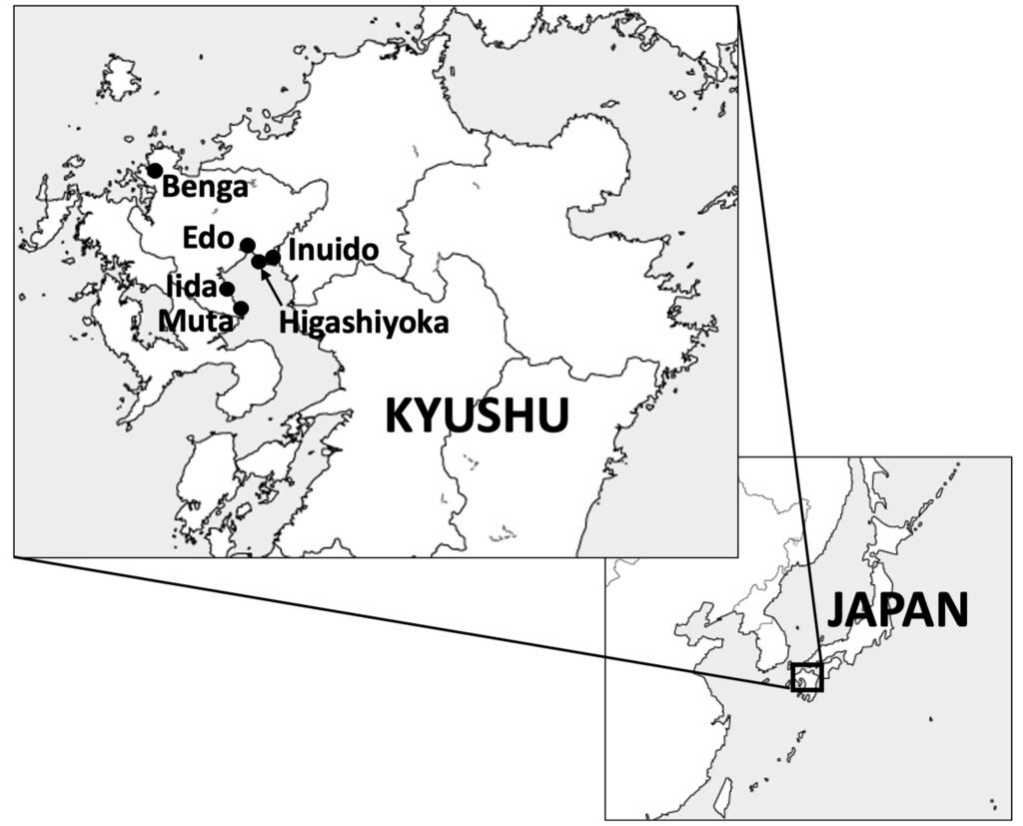
Map of census sites. *Suaeda* plants were surveyed at the following sites: *S.glauca* in Benga, *S.japonica* in Edo and Higatayoka, *S.malacosperma* in Inuido and *S.maritimaasiatica* in Iida and Muta.

Field investigations were conducted at the following six census sites in Saga Prefecture: Benga, Imari City (*S.glauca*); Iida, Kashima City (*S.maritimaasiatica*); Muta, Tara Town (*S.maritimaasiatica*); Inuido, Saga City (*S.malacosperma*); Edo, Saga City (*S.japonica*); and Higashiyoka, Saga City (*S.japonica*) (Fig. 1). Amongst them, Benga faces the Genkai Sea, i.e. areas between the Japan Sea and the East China Sea and the other five sites are located alongthe coastline of the Ariake Sea. The investigations were conducted at two-week intervals from April to November 2015 and monthly from December 2015 to December 2016.

At each census site, insects on *Suaeda* plants were collected by an approximately five-minute sweeping on each census date (qualitative survey). In 2016, three quadrats (1 m × 1 m) were set in *Suaeda* communities and insects inhabiting there were visually investigated in each quadrat (quantitative survey) and collected by net sweeping. Collected insects were kept either as dried specimens or in 99% ethanol for future DNA analyses.

Inventory data of insects on each *Suaeda* species were based on both qualitative and quantitative surveys and the seasonal occurrence data of major species were on the qualitative survey.

## Results and Discussion

### Insect fauna

Throughout the field surveys, we found 18 insect species belonging to five orders from *Suaeda* (Table 1).

In Orthoptera, *Gampsocleisbuergeri* de Haan (Tettigoniidae) and *Euparatettixinsularis* Bey-Bienko (Tetrigidae) were found respectively on *S.glauca* (in 2015) and *S.malacosperma*. Amongst them, *G.buergeri* is distributed in western Honshu and northern Kyushu, Japan and *E.insularis*is is in Honshu, Shikoku, Kyushu, the Izu Islands, the Ogasawara Islands and Korean Peninsula (Ichikawa et al. 2006). Both species are polyphagous and not specialists of *Suaeda* plants.

**Figure 2.**
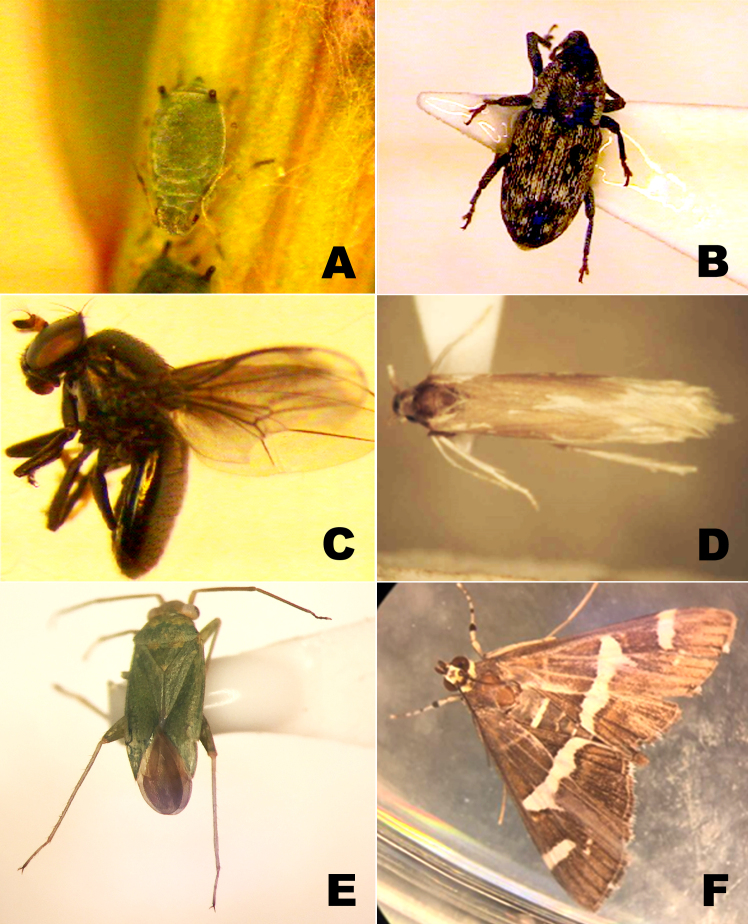
Insects found on *Suaeda* plants. **A** A nymph of *Aphis* sp. (Hemiptera: Aphididae) found on *S.maritimaasiatica* in Iida; **B** An adult of *Barisscolopacea* (Coleoptera: Curculionidae) found on *S.glauca* in Benga; **C** An adult of *Clanoneurum* sp. (Diptera: Ephidridae) found on *S.japonica* in Edo; **D** An adult of *Coleophoradeviella* (Lepidoptera: Coleophoridae) found on *S.japonica* in Edo; **E** An adult of Orthotylus (Melanotrichus) parvulus (Hemiptera: Miridae) found on *S.maritimaasiatica* in Muta; and **F** An adult of *Spoladearecurvalis* (Lepidoptera: Crambidae) found on *S.glauca* in Benga.

In Hemiptera, *Aphis* sp. (Aphididae) was found on all four *Suaeda* species surveyed (Fig. 2A). This species is probably undescribed and a specialist of *Suaeda* plants (Y. Matsumoto, personal communication). Orthotylus (Melanotrichus) parvulus (Miridae) was found on *S.maritimaasiatica* and *S.japonica* (Fig. 2E). This species is associated with *S.maritimaasiatica* and *Salicorniaeuropaea* (Amaranthaceae) and is distributed in the Palearctic Region, but in Japan, it was only known from Tsushima Island and Hyogo Prefecture, Honshu (Yasunaga et al. 2001, Yasunaga et al. 2016, Shishido and Yasunaga 2016). We newly report this species from Kyushu, as mentioned earlier and *S.japonica* is a new host record for this species.

In Coleoptera, *Barisscolopacea* Germar (Curculionidae) was found on *S.japonica* and *S.glauca* in 2015 (Fig. 2B). This weevil is distributed in Honshu, Shikoku, Kyushu, Tsushima, Amami-Oshima and Kuroshima in Japan, as well as in Korea and China (Yoshihara 2016). In western parts of Japan, *B.scolopacea* is associated with Amaranthaceae and induces galls on *Achyranthes* sp., *Chenopodium* spp. and *Dysphaniaanthelmintica* (L.) Mosyakin et Clemants (= *Ambrinaanthelmintica* (L.) Spach) (Yoshihara 2016). In this study, we did not find galls on *Suaeda* plants. As we found stem galls on *Atriplexpatens* (Litv.) Iljin (Amaranthaceae) growing close to *Suaeda*, the weevil is possibly responsible for them. Three widely distributed species of Coccinellidae in Japan were found on *S.japonica*, namely *Harmoniaaxyridis* (Pallas), *Coccinellaseptempunctata* L. and *Propyleajaponica* (Thunberg). As these coccinellids are predatory species (Sakamoto 2018), they probably visit *Suaeda* plants to feed on aphids and other insects. Furthermore, *Medythianigrobilineata* (Motschulsky) (Chrysomelidae) was found on *S.malacosperma* and *Eobiacinereipennis* (Motschulsky) (Oedemeridae) was on *S.glauca* (in 2015). *Medythianigrobilineata* is distributed in Hokkaido, Honshu, Shikoku, Kyushu, Sado, Tsushima, the Goto Islands, the Korean Peninsula, China and eastern Siberia and is known to feed on Fabaceae (Morimoto and Hayashi 1986). As we found wild fabaceous plants near the census site of *S.malacosperma*, the species may accidentally visit *S.malacosperma* from them. *Eobiacinereipennis* is distributed in Hokkaido, Honshu, Shikoku, Kyushu, Izu Islands, Amami Islands, Ryukyus and Korea. Although this species is a flower-visiting species inhabiting seasides (Morimoto and Hayashi 1986), it was collected in June which is not the flowering season of *S.glauca*. For this reason, this species probably visited *S.glauca* by chance while visiting the flowers of other plants growing around *S.glauca*.

In Diptera, *Clanoneurum* sp. (Ephidridae) was found on *S.japonica* and *S.maritimaasiatica* (Fig. 2C) and two species of Syrphidae, namely *Epistrophebalteata* de Geer and *Metasyrphusnitens* Zetterstedt, were found on *S.japonica*. The genus *Clanoneurum* is newly reported from Japan in this paper. This genus contains four species worldwide (GBIF Secretariat 2019). Further taxonomic studies are needed to confirm whether the species found in this study is undescribed. As the syrphids are found in October, the flowering season of *S.japonica*, they might visit the flowers of the plant. The pollination ecology of these *Suaeda* plants is an important study subject in the future.

In Lepidoptera, *Coleophoradeviella* Zeller (Coleophoridae) was found on *S.japonica* and *S.maritimaasiatica* (Fig. 2D). This species is known in the western Palaearctic Region (from Spain to southern Russia) and is associated with several species of Amaranthaceae including *S.maritima* (Anikin 1988, Ellis 2020). This is the first record of *C.deviella* from the eastern Palearctic Region. Two species of polyphagous Noctuidae were found in this study; *Sarcopoliailloba* (Butler) was on *S.glauca* and *Spodopteralitura* (Fabricius) was on *S.glauca* and *S.japonica*. Both species are polyphagous species (Yoshimatsu et al. 2011). In addition, *Spoladearecurvalis* (Fabricius) (Crambidae) was found on *S.japonica*, *S.glauca* and *S.maritimaasiatica* (Fig. 2F). This species is polyphagous and known to be associated with Amaranthaceae and some other plants (Yoshimatsu and Miyata 2011). *Spoladearecurvalis* was previously collected over the East China Sea and, in Kyushu, large numbers of individuals have been collected in autumn along the coast, suggesting its long-distance migration habit (Yoshimatsu and Miyata 2011). In addition, larvae of an unidentified species of Geometridae were found on *S.japonica* and *S.maritimaasiatica*.

### Seasonal occurrence

Amongst insects found on *Suaeda* species, the seasonal occurrence of *Aphis* sp., *Clanoneurum* sp. and *C.deviella* was investigated in the quantitative survey in 2016 by counting individuals of *Aphis* sp., leaves mined by *Clanoneurum* sp. and larval cases formed by *C.deviella* on plants, respectively.

**Figure 3.**
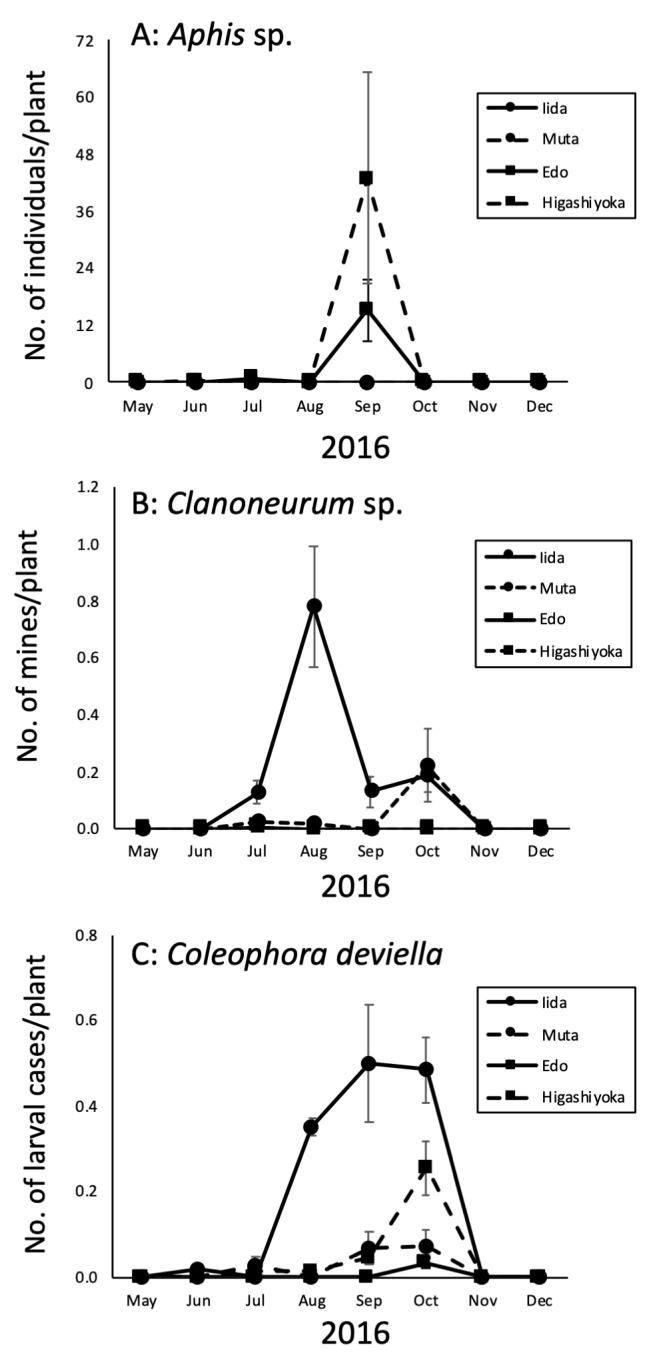
Seasonal changes in densities of major insects on *Suaeda*. **A** Seasonal changes in the number of *Aphis* sp. individuals (per plant) on *Suaeda* plants in 2016; **B** Seasonal changes in the number of mines (per plant) produced by *Clanoneurum* sp. larvae on *Suaeda* plants in 2016 and **C** Seasonal changes in the number of larval cases (per plant) formed by *Coleophoradeviella* on *Suaeda* plants in 2016.

In Edo and Higashiyoka, the number of *Aphis* sp. individuals peaked in September (Fig. 3A). In addition, another small peak was detected in July in Edo. As no individuals were found in May and from October to December, this species may exhibit host alternation, but hosts, other than *Suaeda*, are not yet known at present.

Mines produced by *Clanoneurum* sp. were found from July to October in Iida and Muta (Fig. 3B). At both census sites, two peaks were found in summer (July or August) and autumn (October). The first peak was larger than the second in Iida and vice versa in Muta. These results suggest that the species is bivoltine.

The number of larval cases formed by *C.deviella* gradually increased from summer to autumn and peaked in September or October in all localities (Fig. 3C), suggesting the univoltine life cycle of this species.

## Conclusions

In this study, we recognised 18 insect species on *Suaeda* plants and investigated seasonal occurrence of several herbivorous species. Amongst the insects, *Aphis* sp. is probably an undescribed species; *O.parvulus* was newly recorded from Kyushu; the genus *Clanoneurum* and *C.deviella* were newly reported from Japan. As mentioned in the Introduction, faunistic studies of insects associated with *Suaeda* is limited worldwide, but our findings indicate diverse fauna of insects, especially halophyte-associated herbivores adapting to intertidal zones.

## Figures and Tables

**Table 1.** Insects found on *Suaeda* species and their feeding habit. Abbreviations of *Suaeda* plants are as follows: SG, *S.glauca*; SJ, *S.japonica*; SMRT, *S.maritimaasiatica*; and SMRC, *S.maracosperma*. P: present on the plant.

Order	Family	Species	Feeding habit	SG	SJ	SMRT	SMRC
Orthoptera	Tettigoniidae	*Gampsocleisbuergeri*	Omnivore	P			
	Tetrigidae	*Euparatettixinsularis*	Herbivore				P
Hemiptera	Aphididae	*Aphis* sp.	Herbivore		P	P	P
	Miridae	Orthotylus (Melanotrichus) parvulus	Herbivore	P	P	P	
Coleoptera	Curculionidae	*Barisscolopacea*	Herbivore	P	P		
	Coccinellidae	*Coccinellaseptempunctata*	Predator		P		
		*Harmoniaaxyridis*	Predator		P		
		*Propyleajaponica*	Predator		P		
	Chrysomelidae	*Medythianigrobilineata*	Herbivore				P
	Oedemeridae	*Eobiacinereipennis*	Herbivore	P			
Diptera	Ephydridae	*Clanoneurum* sp.	Herbivore		P		P
	Syrphidae	*Metasyrphusnitens*	Herbivore		P		
		*Epistrophebalteata*	Herbivore		P		
Lepidoptera	Coleophoridae	*Coleophoradeviella*	Herbivore		P	P	
	Noctuidae	*Sarcopoliailloba*	Herbivore	P			
	Noctuidae	*Spodopteralitura*	Herbivore	P	P		
	Crambidae	*Spoladearecurvalis*	Herbivore	P		P	
	Geometridae	gen. sp. (unidentified)	Herbivore		P		P
Number of insect species found				7	12	4	5
